# The effect of body weight on altered expression of nuclear receptors and cyclooxygenase-2 in human colorectal cancers

**DOI:** 10.1186/1475-2891-6-20

**Published:** 2007-09-03

**Authors:** Barbara Delage, Anne Rullier, Maylis Capdepont, Eric Rullier, Pierrette Cassand

**Affiliations:** 1 Laboratoire Alimentation et Cancerogenese Colique, Unite de Nutrition et Signalisation Cellulaire, Universite Bordeaux1, France; 2 Departement de Pathologie, Hopital Pellegrin, Bordeaux, France; 3 Departement de Chirurgie Digestive, Hopital Saint-André, Bordeaux, France

## Abstract

**Background:**

Epidemiological studies on risk factors for colorectal cancer (CRC) have mainly focused on diet, and being overweight is now recognized to contribute significantly to CRC risk. Overweight and obesity are defined as an excess of adipose tissue mass and are associated with disorders in lipid metabolism. Peroxisome proliferator-activated receptors (PPARs) and retinoid-activated receptors (RARs and RXRs) are important modulators of lipid metabolism and cellular homeostasis. Alterations in expression and activity of these ligand-activated transcription factors might be involved in obesity-associated diseases, which include CRC. Cyclooxygenase-2 (COX-2) also plays a critical role in lipid metabolism and alterations in COX-2 expression have already been associated with unfavourable clinical outcomes in epithelial tumors. The objective of this study is to examine the hypothesis questioning the relationship between alterations in the expression of nuclear receptors and COX-2 and the weight status among male subjects with CRC.

**Method:**

The mRNA expression of the different nuclear receptor subtypes and of COX-2 was measured in 20 resected samples of CRC and paired non-tumor tissues. The association between expression patterns and weight status defined as a body mass index (BMI) was statistically analyzed.

**Results:**

No changes were observed in PPARγ mRNA expression while the expression of PPARδ, retinoid-activated receptors and COX-2 were significantly increased in cancer tissues compared to normal colon mucosa (*P *≤ 0.001). The weight status appeared to be an independent factor, although we detected an increased level of COX-2 expression in the normal mucosa from overweight patients (BMI ≥ 25) compared to subjects with healthy BMI (*P *= 0.002).

**Conclusion:**

Our findings show that alterations in the pattern of nuclear receptor expression observed in CRC do not appear to be correlated with patient weight status. However, the analysis of COX-2 expression in normal colon mucosa from subjects with a high BMI suggests that COX-2 deregulation might be driven by excess weight during the colon carcinogenesis process.

## Background

Approximately 5% of individuals will develop colorectal carcinoma during their lifetime [1]. This disease typically progresses from adenomatous polyps and dysplastic polyps to invasive carcinoma [2,3]. CRC seems particularly susceptible to specific nutritional factors and dietary habits (for review [4]). Indeed, excessive consumption of calories from fat is thought to be largely responsible for the increasing incidence of CRC in western countries. Moreover, overweight and obesity status associated with high body mass indices have been correlated with a higher risk of developing CRC [5,6]. Nevertheless, the mechanisms of why and how excess weight increases cancer risk are only slowly emerging. One proposed mechanism is the rise of insulin resistance resulting in hyperinsulinemia that can cause growth-promoting effects [7].

Hyperinsulinemia as well as hyperlipidemia, hypertension, overweight and type II diabetes are metabolic disorders which might be caused by alterations in the homeostasis of the metabolism of fatty acids [8]. These obesity-related symptoms could affect the integrity of colon tissue homeostasis and therefore be involved in CRC etiology [9]. Anti-diabetic treatments initially used for improving parameters such as insulin sensitivity have been shown to be able to inhibit colon carcinogenesis in rodent models [10,11] and to promote differentiation of colon cancer cells [12]. Moreover, concomitant suppression of hyperlipidemia and polyp formation were observed in APC-deficient mice treated with insulin-sensitizing drugs called thiazolidinediones (TZD) [13]. In such a context, PPAR family members initially recognized for their involvement in regulating fat metabolism and adipogenesis have emerged as attractive targets for therapeutic approaches for obesity and CRC. Indeed, PPARγ agonists including anti-diabetic agents, polyunsaturated fatty acids as well as non-steroidal anti-inflammatory drugs (NSAID) [14,15] have been demonstrated to affect proliferation and differentiation in cancer cell lines [16]. Moreover, both anti-proliferative effects of PPARγ observed *in vitro *[12] and inactivating mutations in the PPARγ gene found in colon tumors [17] provide evidence for a tumor suppressor function. This is also supported by the finding that an increased risk of polyp occurrence in colon mucosa was found to be significantly associated with a decrease in PPARγ mRNA expression [18]. Another isotype, PPARδ, may also play an important role in the process of colon carcinogenesis since it has been efficiently targeted by hypolipidemic and hypoglycemic drugs [19]. However, PPARδ might display distinct functions in lipid metabolism and colon carcinogenesis. Indeed and in contrast to PPARγ, PPARδ was found frequently overexpressed in colon cancer cells [20] and tumors of chemically-initiated animals [21]. Nevertheless, the role of PPARδ in colonic epithelium stays unclear due to conflicting evidence [22].

The transcriptional activity of PPAR depends on the presence of the retinoic X receptor (RXR), activated by 9-cis retinoic acid (9-cis RA). Heterodimerization with RXR is essential for the activity of all class II nuclear receptors [23] and explains how fatty acids and retinoids control lipid metabolism [24]. The active forms of vitamin A, 9-cis RA and all-trans retinoic acid (atRA), also exhibit anti-tumoral properties in many tissues mainly through RXR and retinoic acid-activated receptor (RAR) binding. Indeed, retinoids have displayed chemopreventive and chemotherapeutic activities with regard to their capacity to induce cell differentiation and apoptosis (for review [25]). RXRα is by far the most prevalent isoform in the colon, while RXRβ and RXRγ are expressed at low levels [26]. All three RAR isotypes, α, β, and γ, are expressed and induced by retinoids in colon cancer cell lines [27]. Alterations in retinoid-activated receptor expression and biological activity have been observed both *in vivo *and *in vitro *[28]. However, potential alterations have been poorly investigated in CRC although they may affect the response of target cells to retinoid and lipid derivatives.

COX-2 is a key enzyme in lipid metabolism and is well-known to convert arachidonic acid to growth-regulating molecules such as prostaglandins. COX-2, activated by growth factors and pro-inflammatory cytokines, has been shown to be overexpressed in several epithelial cancers including CRC [29,30]. This enzyme might mediate the promotion of colon carcinogenesis by metabolic disorders and inflammation and several lines of evidence indicate that COX-2 might be regulated by PPARγ [31] and RARβ activation in cancer cells [32,33] although the mechanism is unclear. It was also shown that deregulations in nuclear receptor expression might promote COX-2 upregulation [34].

In the current report, our interest was (i) to evaluate alterations in the expression of the different nuclear receptors and COX-2 in several colon cancer specimens from patients undergoing surgery to remove tumors and (ii) to clarify whether or not the expression of nuclear receptors and COX-2 was affected by the weight status of patients with CRC.

## Methods

### Patients and samples

Tumor samples and normal adjacent tissue microscopically confirmed to be free of cancer were obtained from 20 patients undergoing surgery for colorectal cancer at the St. André Hospital (Bordeaux, France) from January 2003 to September 2004. Following surgery, samples from tumor and adjacent normal mucosa were frozen in liquid nitrogen and stored at -80°C for subsequent RNA extraction. All included patients were men without neoadjuvant treatment. Patients suffering from familial cancer syndromes were excluded. Information concerning age, BMI (weight (kg)/height (m)^2^), tumor site, pTNM and Dukes stage are indicated in Table 1. BMI was found not to be correlated with height in this population (*r *= 0.07, n = 20), but strongly correlated with weight (*r *= 0.89, n = 20). Segregation of patients into BMI groups corresponded to the following categorizations: 18.5–25 kg/m^2 ^(healthy weight), ≥ 25 kg/m^2 ^(excess weight group including overweight/pre-obese/obese subjects).

**Table 1:** Summary of clinical and pathological data

Case n°	Age	BMI	T^a^	N^a^	M^a^	Dukes^b^	Site
1	80	20.83	4	1	x	C	asc. colon
2	80	29.75	3	1	x	C	asc. colon
3	72	38.75	3	1	x	C	rectum
4	82	31.41	3	0	x	B	rectum
5	81	21.47	3	0	x	B	sigmoid
6	69	25.34	3	0	x	B	asc. colon
7	56	24.68	3	1	1	D	asc. colon
8	66	21.91	3	0	x	B	asc. colon
9	69	24.34	4	1	x	C	rectum
10	86	24.91	4	1	1	D	asc. colon
11	87	23.03	3	1	x	C	rectum
12	66	27.68	4	0	x	B	sigmoid
13	73	28.13	4	1	x	C	sigmoid
14	71	34.60	3	1	x	C	rectum
15	60	30.24	2	0	1	D	sigmoid
16	61	26.12	3	0	x	B	sigmoid
17	41	18.73	4	1	x	C	asc. colon
18	85	25.95	3	1	x	C	asc. colon
19	72	29.06	2	0	1	D	des.colon
20	76	22.72	3	1	1	D	rectum

^a ^According to the TNM classification : T, primary tumor; N, regional lymph nodes; M, metastasisb According to the Dukes classification

^b ^According to the Dukes classification

### RNA extraction and reverse transcription (RT)

Total RNA was extracted from colon tissue samples using the RNAgents Total RNA Isolation System kit according to the manufacturer's instructions (Promega, Charbonnières, France). Reverse transcription was as follows: 2 μg of total RNA was mixed with RNasin (1 U/μL, Promega, Charbonnières, France) and DNase I (0.5 U/μL, Roche Diagnostics, Meylan, France) and incubated 15 min at 37°C. Reverse primers (0.75 μM of each) were added and incubated for 10 min at 70°C. ImProm-II™ 5× reaction buffer (1×, Promega, Charbonnières, France), MgCl_2 _(2.5 mM, Promega, Charbonnières, France), dNTP (0.5 mM each one, Roche Diagnostics, Meylan, France) and ImProm-II™ Reverse Transcriptase (Promega, Charbonnières, France) were added for 1 hr at 42°C. The total volume was 20 μL and each target mRNA was co-reverse transcribed with β2-microglobulin mRNA.

### Real-time Polymerase Chain Reaction (PCR)

Real-time quantitative PCR involving LightCycler™ technology (Roche Diagnostics, Mannheim, Germany), was performed according to the protocol recommended by the manufacturer and previously described [34]. SYBR green I fluorescence dye was sufficiently sensitive to accurately detect amplified products from all target cDNA (PPARδ, PPARγ, RARα, RXRα and COX-2) except for RARβ and RARγ amplified product detected using dual-labeled and specific TaqMan fluorogenic probes. Quantification data were analyzed using the LightCycler Relative Quantification Software (Roche Diagnostics, Mannheim, Germany). The software provides a crossing point (Cp), defined as the PCR cycle number, function of the log of the DNA concentration (in ng). A standard curve is a plot of the Cp versus the amount of initial cDNA used for amplification. Standard curves were used to estimate the concentration of both the target and the reference gene in each sample. This software provides a calibrator-normalized relative quantification including PCR efficiency correction considering then the difference existing between amplification efficiencies of reference and target cDNAs. cDNA from tissue samples from patient A was arbitrarily chosen to be the calibrator. The cDNA calibrator was used in all experiments. Results are expressed as the target:reference ratio divided by the target:reference ratio of the calibrator. Primers and fluorogenic probes were purchased from Proligo France (Paris, France). Each probe was synthesized with the fluorescent reporter dye FAM (6-carboxy-fluorescein) attached to the 5'-end and a quencher dye TAMRA (6-carboxy-tetramethyl-rhodamine) to the 3'-end. Specificity of primers was validated through the verification of RT-PCR product specificity. RT-PCR products were subjected to analysis by electrophoresis on a 1.5% agarose gel and resulted in a single product with the desired length (β2-microglobulin, 112 bp; PPARδ, 139 bp; PPARγ, 144 bp; RARα, 235 bp; RARβ, 133 bp; RARγ, 167 bp; RXRα, 142 bp; COX-2, 130 bp). The identity of amplified products were assessed by sequencing with a Dye Terminator Reaction Cycle Kit (Perkin-Elmer, Norwalk, CT) and were analyzed on an ABI PRISM™ 377 automated DNA sequencer (Perkin-Elmer). The forward and reverse primer sequences and the probes were as follows:

*β2-microglobulin*: sense 5' CTTGGGCTGTGACAAAGTC 3', antisense 5' GTCTTTC-AGCAAGGACTGG 3', Taqman probe 5' (6-Fam)TGGTTCACTCGGCAGGCATAC-TC(Tamra) 3';

*PPARδ*: sense 5'GGGAGAGGTCTGTGTAGCTGCTG 3', antisense 5' ATGGAGCA-GCCACAGGAGGAAGCC 3';

*PPARγ*: sense 5' CGGATGGCCACCTCTTTGCTC 3', antisense 5' GGCGAGGGCG-ATCTTGACAGG 3';

*RARα*: sense 5' ACGTTGTTCTGAGCTGTTGTTCGTA 3', antisense 5' CTGCCAGT-ACTGCCGACTGC 3';

*RARβ*: sense 5' AG-GCTTGCTGGGTCGTCTTT 3', antisense 5' CCTTCTCAGTGC-CATCTGCTTAAT 3', Taqman probe 5' (6-Fam)AGACCGCCAGGACCTTGAGGA-ACCGA(Tamra) 3';

*RARγ*: sense 5' GCAAAGACAAGGTCTGT-GAG 3', antisense 5' GACCAGATCAC-TCTGCTCAAAGC 3', Taqman probe 5' (6-Fam)TATCCTGATGCTGCGTATCTGC-ACAAGGT(Tamra);

*RXRα*: sense 5' GAGCAGCTTATTCCAGCCTGCC 3', antisense 5' CGACCCTGTC-ACCAACATTTGC 3';

*COX-2*: sense 5'TGGTGCCTGGTCTGATGAT 3', antisense 5' GCCTGCTTGTCTG-GAACAAC 3'

### Statistical methods

Statistical analyses were carried out using the Windows SPSS^® ^9.0 software package. Associations between clinicopathological variables (age, TNM and Dukes stage (Dukes B *vs. *C *vs. *D), BMI (BMI < 25 *vs. *BMI ≥ 25), tumor site (descendant/sigmoid colon/rectum *vs. *ascendant colon)) were assessed by Spearman's correlation coefficient test. Associations between mRNA expression levels were tested for correlation by Spearman's test and mRNA levels were compared with regard to clinicopathological features. Specifically, comparison of mRNA expression levels in healthy tissue with regard to BMI and tumor site was performed using the Mann-Whitney U test. The Kruskal-Wallis test was used to assess for significant differences in mRNA expression with regard to Dukes stage (Dukes B *vs. *C *vs. *D). The significance of differences in mRNA expression levels between healthy mucosa and tumor tissue was evaluated using the Wilcoxon-test. A *P *value < 0.05 was considered as significant.

## Results

Samples of CRC specimens and adjacent non-neoplastic colonic mucosa were collected from patients undergoing surgery. Pertinent clinical and pathological data are listed in Table 1. All patients were men with a median age of 72 years old (range 41–87). Six patients (30%) had Dukes' B tumors, nine patients (45%) were classified as Dukes' C or as Dukes' D (25%). Eleven patients (55%) had a BMI greater than 25, and were designated as overweight. Four of these had a BMI value above 30, corresponding to obesity status. No correlation was found between age, BMI, and tumor classification.

Nuclear receptors and COX-2 were detectable by quantitative real-time RT-PCR in all normal-looking tissue and tumor samples. The median mRNA expression values are shown in Table 2. The median relative PPARγ expression level in tumors remained unchanged as compared to normal mucosa. Indeed, among 20 investigated cancer tissue samples, PPARγ increased between 1.5- and 4-fold in 35% (n = 7), while we noted a 1.5- to 6-fold decrease in 25% (n = 5). No changes were observed in the remaining 40% (n = 8). In contrast, the expression of PPARδ in tumors was significantly upregulated (1.54 *vs. *1.30, *P *= 0.001) relative to normal mucosa. All retinoid nuclear receptors were also upregulated in tumor tissues compared to healthy mucosa (n = 20, *P *< 0.001). Expression levels were increased by the following percentages: RXRα 26.7%, RARα 27.4%, RARβ 54.9%, and RARγ 149.6%. COX-2 mRNA expression was multiplied by 8.5 between normal and tumor tissues (*P *< 0.001). Relationships between nuclear receptor and COX-2 mRNA expression were tested statistically and listed in Tables 3 and 4 (see also additional file 1). Further combined analysis of receptor and COX-2 mRNA expression levels with regard to Dukes' stages and tumor localisations did not display any significant statistical difference. COX-2 expression was not correlated with tumor stages and localisations (data not shown), but was associated with RARα and RARβ mRNA expression in tumor tissue (Table 4).

**Table 2:** Nuclear receptor and COX-2 mRNA expression in colorectal tumors

Gene	n	Normal colon mucosa		Tumor tissue		p^a^
				
		Median	Range	Median	Range	
PPARγ	20	0.96	(0.35–3.19)	1.04	(0.28–4.71)	n.s.
PPARδ	20	1.30	(0.69–2.34)	1.54	(0.40–4.96)	0.001
RXRα	20	0.75	(0.20–1.25)	0.95	(0.21–3.49)	<0.001
RARα	20	1.13	(0.69–1.88)	1.44	(0.38–3.93)	<0.001
RARβ	20	1.42	(0.12–5.39)	2.20	(0.18–10.82)	<0.001
RARγ	20	1.37	(0.49–9.52)	3.42	(0.83–15.80)	<0.001
COX-2	20	1.50	(0.44–6.75)	12.80	(0.50–560.5)	<0.001

Statistical analyses were performed using Wilcoxon-test

n.s. not significant

a Wilcoxon test

**Table 3:** Associations between nuclear receptor and COX-2 expression in healthy colorectal mucosa

	PPARγ	PPARδ	RXRα	RARα	RARβ	RARγ	COX-2
PPARγ	1.000	0.104 (n.s.)	0.324 (0.012)^a^	0.107 (n.s.)	0.330 (0.010)	-0.017 (n.s.)	0.170 (n.s.)
PPARδ		1.000	0.179 (n.s.)	0.277 (0.032)	-0.001 (n.s.)	-0.119 (n.s.)	-0.145 (n.s.)
RXRα			1.000	0.255 (0.049)	0.233 (n.s.)	0.111 (n.s.)	0.003 (n.s.)
RARα				1.000	0.2580 (0.046)	-0.112 (n.s.)	0.134 (n.s.)
RARβ					1.000	-0.294 (0.023)	0.173 (n.s.)
RARγ						1.000	0.261 (0.044)
COX-2							1.000

**Table 4:** Associations between nuclear receptor and COX-2 expression in colorectal tumors

	PPARγ	PPARδ	RXRα	RARα	RARβ	RARγ	COX-2
PPARγ	1.000	0.390 (0.002)^a^	0.364 (0.004)	0.408 (0.001)	0.105 (n.s.)	0.436 (<0.001)	-0.191 (n.s.)
PPARδ		1.000	0.620 (<0.001)	0.773 (<0.001)	0.571 (<0.001)	0.601 (<0.001)	0.407 (n.s.)
RXRα			1.000	0.577 (<0.001)	0.438 (<0.001)	0.425 (0.001)	0.161 (n.s.)
RARα				1.000	0.581 (<0.001)	0.538 (<0.001)	0.291 (0.024)
RARβ					1.000	0.434 (0.001)	0.453 (<0.001)
RARγ						1.000	0.202 (n.s.)
COX-2							1.000

n.s. not significant

a correlation coefficient with the p value from Spearman's test in parentheses

Differences in expression of nuclear receptors and COX-2 between normal and tumor tissues (Table 2) were also observed when patients were segregated into groups with low and high BMI (BMI < 25 *vs. *BMI ≥ 25) (Tables 5 and 6). We also compared the expression of nuclear receptors and COX-2 in healthy mucosa regarding the BMI of patients (Table 7). Statistics revealed that COX-2 expression is significantly increased in the normal-looking mucosa from patients with the highest BMI (Table 7).

**Table 5:** Nuclear receptor and COX-2 mRNA expression in colorectal tumors of patients with BMI < 25

Gene	n	Healthy colon mucosa		Tumor tissue		p^a^
				
		Median	Range	Median	Range	
PPARγ	9	1.00	(0.65–3.19)	1.17	(0.35–3.67)	n.s.
PPARδ	9	1.35	(0.69–2.34)	1.55	(0.57–4.96)	0.012
RXRα	9	0.76	(0.37–1.20)	1.09	(0.54–1.84)	<0.001
RARα	9	1.09	(0.69–1.85)	1.58	(0.95–3.93)	<0.001
RARβ	9	1.53	(0.52–2.49)	2.78	(0.18–8.49)	0.001
RARγ	9	1.21	(0.76–3.68)	4.49	(0.91–9.68)	<0.001
COX-2	9	1.11	(0.51–3.48)	12.40	(0.50–560.46)	<0.001

**Table 6:** Nuclear receptor and COX-2 mRNA expression in colorectal tumors of patients with BMI ≥ 25

Gene	n	Healthy colon mucosa		Tumor tissue		p^a^
				
		Median	Range	Median	Range	
PPARγ	11	0.95	(0.35–2.11)	0.97	(0.28–4.71)	n.s.
PPARδ	11	1.28	(0.72–2.00)	1.52	(0.40–4.49)	0.022
RXRα	11	0.64	(0.20–1.25)	0.85	(0.21–3.49)	0.047
RARα	11	1.17	(0.72–1.88)	1.44	(0.38–3.72)	0.018
RARβ	11	1.30	(0.12–5.39)	2.12	(0.24–10.82)	0.014
RARγ	11	1.60	(0.49–9.52)	3.17	(0.83–15.80)	<0.001
COX-2	11	2.93	(0.44–6.75)	13.20	(0.85–166.86)	<0.001

**Table 7:** Comparison between nuclear receptor and COX-2 mRNA expression in healthy colorectal mucosa from patients with BMI < 25 or with BMI ≥ 25

Gene	BMI < 25			BMI ≥ 25			p^a^
			
	n	Median	Range	n	Median	Range	
PPARγ	9	1.00	(10.65–3.19)	11	0.95	(0.35–2.11)	n.s.(0.882)
PPARδ	9	1.35	(0.69–2.34)	11	1.28	(0.72–2.00)	n.s.(0.882)
RXRα	9	0.76	(0.37–1.20)	11	0.64	(0.20–1.25)	n.s.(0.095)
RARα	9	1.09	(0.69–1.85)	11	1.17	(0.72–1.88)	n.s.(0.824)
RARβ	9	1.53	(0.52–2.49)	11	1.30	(0.12–5.39)	n.s.(0.370)
RARγ	9	1.21	(0.76–3.68)	11	1.60	(0.49–9.52)	n.s.(0.230)
COX-2	9	1.11	(0.51–3.48)	11	2.93	(0.44–6.75)	0.002

Statistical analyses were performed using Mann-Whitney test

n.s. not significant

a Mann-Whitney test

## Discussion

Nuclear receptors are involved in many cellular processes from embryonic development to cell death. Dysfunction of nuclear receptor signaling can lead to proliferative and metabolic diseases such as cancer and obesity. In the current report, we assessed the mRNA expression levels of nuclear receptors and COX-2 in 20 CRC specimens and sought a possible relationship with patient's weight status defined by BMI ranging from 18.7 to 38.7.

PPARγ constitutes the most extensively studied of the three PPAR subtypes (α, β, γ) since its function relates to lipid metabolism as well as cell differentiation, apoptosis and cancer. PPARγ can be activated by certain lipids and derivatives and by anti-diabetic agents. Activated PPARγ has been shown able to stimulate differentiation and apoptosis in cancer cells from various origins [35-38]. Nevertheless, in contrast with results generated *in vitro*, data concerning PPARγ expression in human cancer specimens raised questions about the anti-neoplastic activity of the receptor *in vivo*. For example, PPARγ was found highly expressed in ovarian carcinoma [39] and its overexpression in pancreatic carcinoma was associated with poor prognosis [40]. By contrast, our data, in agreement with others [41], showed PPARγ expression globally unchanged in CRC compared with adjacent normal tissues, although Dubois et al. [42] found a marked increase of PPAR mRNA expression in four CRC samples and in different colon cancer cell lines. Discrepancies might be attributed to germline mutations in the adenomatous polyposis gene (APC). Indeed, PPARγ has been involved in increasing resistance towards carcinogens by preventing the accumulation of β-catenin, which is regulated by APC. However, PPARγ functions are lost when *APC *is mutated [43]. Another report has shown that deregulated APC/β-catenin indirectly induced aberrant PPARγ overexpression [44], explaining previous experimental data in *APC*^Min^/+ mice showing a promoting effect of PPARγ on carcinogenesis [45]. This has relevance for humans because mutations in the tumor suppressor gene *APC *are the initiating event in about 85% of sporadic CRC. Therefore, APC status could dramatically affect expression and function of PPARγ and the steady-state levels of PPARγ reported here do not exclude loss of PPARγ transcriptional activity due to somatic mutations [17], alterations in intracellular distribution [46], post-translation modifications [47] or inhibition by PPARδ [48].

Like PPARγ, PPARδ gene expression is detected in the colon and the receptor can be activated by fatty acids and derivatives. Herein, we reported an elevated level of PPARδ (~18%) in CRC. Upregulation of PPARδ gene expression might be attributed to deregulation in the APC/β-catenin pathway since PPARδ is considered to be a downstream target gene [20]. Increased levels of PPARδ expression have already been observed in rodent colorectal tumors and in primary human colorectal adenocarcinomas [20,21]. Nevertheless, PPARδ function remains elusive, with data showing that PPARδ was dispensable for polyp formation [49]. Our data and others suggested a contribution of PPARδ in the carcinogenesis process [16,50] while Marin et al. [22] described that agonist-activated PPARδ protects against cancer development. As for PPARγ, the integrity of the tumor suppressor APC might be essential to guarantee PPARδ normal function.

Critical to the transcriptional activity of PPARs is the ability to form a complex with RXR and bind to DNA. Synthetic ligands of RXRα were shown to exhibit insulin-sensitizing activity [51,52] and to act synergistically with PPARγ ligands to enhance PPARγ/RXRα-mediated transactivation [31]. In addition, a positive correlation in healthy mucosa was found between RXRα and PPARγ supporting the idea of a tight relationship in the regulation of the expression of these receptors. While no change in RXRα expression level was previously noted in 17 patients with CRC [41], our data revealed a significant increase in tumor versus normal tissue. A similar upregulation was also observed in human esophageal [53], breast [54] and hepatocellular carcinomas [32]. However, little is known about the function of RXRα in colon tumorigenesis and our results do not rule out the possibility of alterations in RXRα functions due to altered localization [55] or inhibitory effect of unliganded RXRα on PPARγ transactivation [56].

Recent data have also suggested that PPARγ anti-tumor activity required a functional RARβ [57]. This implies that PPARγ function may be affected by alterations in the retinoid pathway. To our knowledge, very few reports have examined the expression of retinoid receptors in CRC. Therefore, we described here the first detailed analysis of nuclear receptor RARα, β and γ mRNA expression in CRC. RARβ has been extensively studied in cancer cells and human carcinomas, and several studies have suggested that it may play a role as a tumor suppressor gene [58-60]. However, our results showed a significant upregulation in the expression of all three RAR isotypes in CRC specimens compared to adjacent normal mucosa. Furthermore, we showed a complex association between the expression of mRNA for RXRα, RARs, and PPARs in cancer tissue, suggesting interactions and cross-talk between these receptors in tumorigenesis. These results demonstrated that alterations are not restricted to a single receptor. Instead, we observed a profound dysregulation of the retinoid pathway in this CRC. Downregulation of mRNA expression of RARβ often observed in cancer cells has been considered as a cellular mechanism to prevent retinoid-induced growth arrest [61,62]. On the other hand, while elevated levels of RAR mRNA expression has also been described in breast, liver and esophageal tumors [53,63,64], mechanism and significance are unknown. Nevertheless, if the expression of RAR correlates with tissue sensitivity to retinoids, our results should be confirmed within a larger number of samples and both the mechanism leading to inappropriate RXR and RAR expression and the response of CRC to retinoids should be investigated.

There are strong correlations between the intake of fatty acids, the establishment of metabolic disorders and an increased risk of developing CRC [65,66]. This suggests the involvement of PPARs and retinoid receptors, activated by fatty acids and derivatives [21] and modulated by metabolic disorders [67] in establishing a link between overweight prevalence and CRC pathogenesis. In the current report, we aimed to clarify whether aberrations in the expression of nuclear receptors may contribute to associate high BMI and CRC. However, alterations in nuclear receptor expression observed in tumors were similar in both patients with low or high BMI. We also investigated COX-2 expression which is involved in cellular responses to lipids and inflammatory processes that favour tumorigenesis by stimulating cell proliferation and angiogenesis [68]. Interestingly, while COX-2 was greatly expressed in CRC as previously shown [69], we also found a significantly increased level of COX-2 expression in normal mucosa from patients with high BMI compared to low BMI patients. Recently, it has been reported that patients with a high risk of developing CRC presented an upregulation of the COX-2 gene in normal-looking colon mucosa [70]. This supports the idea that COX-2 deregulation might be an early event in the process of carcinogenesis. Nevertheless, although previous reports showed COX-2 regulation by nuclear receptors [31,71], very few associations were found in our study between COX-2 and nuclear receptor expression. In conclusion, our study described altered expression of nuclear receptors in CRC specimens. Further studies are warranted in order to determine the underlying mechanism leading to altered expression of PPARs and retinoid-activated receptors and the significance of such alterations. Moreover, alterations in nuclear receptor expression were independent of the weight status of patients. Nevertheless, COX-2 might be one early target influenced by excess weight and associated metabolic disorders and consequently might affect nuclear receptor expression and activation.

## Authors' contributions

BD and PC designed the research plan; AR and ER provided and analyzed specimens (histopathology); BD performed research; MC performed statistical analyses; BD and PC analyzed data; and BD wrote the paper. All authors read and approved the final manuscript.

## Supplementary Material

Additional file 1 Associations between mRNA expression levels of PPAR, RXR and RAR subtypes in CRC. CC : correlation coefficient; p : p value based on Spearman's test. The figure provided represents the statistical analyses of the correlations between nuclear receptor expression in colorectal tumors. Click here for file
