# Supplementary material for: The effect of body weight on altered expression of nuclear receptors and cyclooxygenase-2 in human colorectal cancers
Source: Nutr J. 2007 Sep 3;6:20. doi: 10.1186/1475-2891-6-20 (PMC2018695; doi:10.1186/1475-2891-6-20)
Supplement: Additional file 1 — Associations between mRNA expression levels of PPAR, RXR and RAR subtypes in CRC. CC : correlation coefficient; p : p value based on Spearman's test. The figure provided represents the statistical analyses of the correlations between nuclear receptor expression in colorectal tumors. [file 1475-2891-6-20-S1.ppt]

## Slide 1
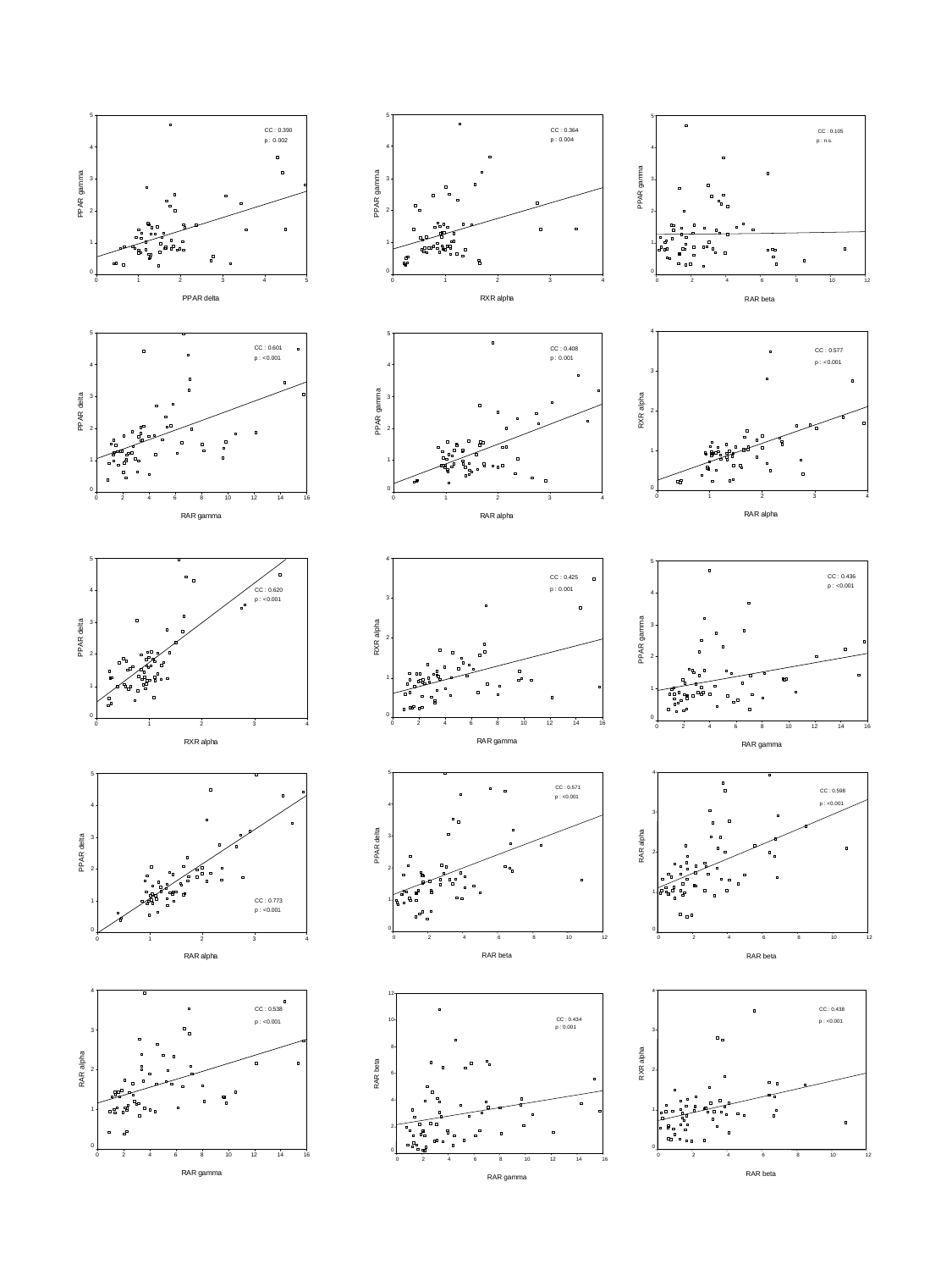

5
CC : 0.105
p : n.s.
4
3
PPAR gamma
2
1
0
0
2
4
6
8
10
12
RAR beta
4
CC : 0.598
p : <0.001
3
RAR alpha
2
1
0
0
2
4
6
8
10
12
RAR beta
5
CC : 0.571
p : <0.001
4
3
PPAR delta
2
1
0
0
2
4
6
8
10
12
RAR beta
4
CC : 0.438
p : <0.001
3
RXR alpha
2
1
0
0
2
4
6
8
10
12
RAR beta
12
CC : 0.434
10
p : 0.001
8
6
RAR beta
4
2
0
0
2
4
6
8
10
12
14
16
RAR gamma
